# Epidemiological Society

**Published:** 1854-09

**Authors:** J. H. Tucker


*Epidemiological Society*—On the use of Vegetable and Mineral Acids in the Treatment, Prophylactic and Remedial, of epidemic disorders of the Bowels. By J. H. TUCKER, ESQ.

The author commenced by alluding to the remarkable, but well-established fact, that in 1849 the cider districts of Herefordshire, Somersetshire, and part of Devonshire, were to a great extent. exempt from the epidemic ravages of cholera, while the disease was raging around. Upon further inquiry it was ascertained that this exemption was confined a good deal to those individuals who drank cider as a common beverage, and that those who partook of malt liquor suffered. He also remarked that. in some parts of France and in Normandy, more particularly where cider is the common beverage. cholera is seldom known to exist; and further, that Switzerland was reported to have been free from its visitation.

Having adduced these and other facts in proof of the prophylactic power of cider. the author expressed his opinion that other vegetable acids would be found of service, such as lemon or orange-juice, and sour wines made from grapes, or even from gooseberries. And as it would be found impossible to supply the whole of London with a sufficient quantity of pure cider. Mr. Tucker suggested that *vinegar* might be found a useful substitute in case of another outbreak of cholera, provided that it could be obtained in a state of purity. In confirmation of his view of the sanative and medicinal virtues of vinegar. the author quoted Hippocrates, who (*de natura meliebri*,) "employed white vinegar medicinally"—Plutarch and Livy, who refer to the use of vinegar by Hannibal, in his passage over the Alps, when he is said to have "softened the rocks with fire and vinegar, an operation which the author facetiously regarded as rather metaphorical than chemical, as the vinegar, swallowed by the troops probably sustained their strength, and thus in effect softened the asperities of their rough way. The author also quoted from Roman history the story that "Scipio Africanus is said to have gained a great battle with a few skins of vinegar," the troops refusing to march until the general had obtained a supply. Cæsar was also reported to mention in his Commentaries the supply of vinegar to the troops; and Mr. Tucker remarked that the drink of the Romans in all their campaigns was vinegar and water, and, sustained by that beverage, they conquered the world. Modern authors (Sir John Pringle, Sir Gilbert Blane, and others) were also quoted in proof of the antiseptic and medicinal qualities of vinegar. The author then proceeded to show that acid drinks were not only preventive, but remedial in epidemic disorders of the bowels. Cases were related, in which not only were exempt from attacks of cholera raging around them who drank long draughts of cider, but a case of cholera was also related, which yielded to the diluted juice of sour apples. The efficacy of the *Mineral Acids*, especially the sulphuric, in diarrhœa, was also advocated by reference to numerous facts and authorities. He also referred to some established facts connected with the spread of epidemic dysentery in the army, showing the efficacy of vegetable acids in that disease.

In conclusion, Mr. Tucker suggested a necessary caution relative to the use of the wretched and unwholesome substitute for vinegar commonly sold in the London shops.

The discussion which followed the reading of the paper, elicited many facts in confirmation of the author's views; and, as to the efficacy of sulphuric acid largely diluted with water, in choleraic diarrhœa, there was not a dissentient voice.

